# Toxic mercury, aquatic ecosystem collapse, and the ancient Lowland Maya

**DOI:** 10.1126/sciadv.aeh3280

**Published:** 2026-11-04

**Authors:** Matthew Neal Waters, Savanna Lee Wooten, Ashley Mark Abrook, Mark Brenner, Jason Hilleary Curtis, Peter Guy Langdon, Roger Manuel Medina-Gonzalez, Savvas Paradeisis-Stathis, Richard Scott Vachula

**Affiliations:** ^1^ Department of Crop, Soil, and Environmental Sciences, Auburn University, Auburn, AL, USA.; ^2^ School of Geography and Environmental Science, University of Southampton, Southampton, UK.; ^3^ Department of Geological Sciences, and Land Use and Environmental Change Institute, University of Florida, Gainesville, FL, USA.; ^4^ Universidad Autónoma de Yucatán, Mérida, México.; ^5^ Department of Geosciences, Auburn University, Auburn, AL, USA.

## Abstract

Environmental impacts endured by the Lowland Maya during the Classic Period (250 to 920 CE) included droughts, deforestation, and soil erosion. Recent studies on mercury (Hg) pollution and cyanotoxins suggest that degraded water quality was also a stressor for the Pre-Columbian Maya. Hg in the Maya region is related to use of both elemental mercury and mineral cinnabar (HgS), the latter used as a red pigment. Questions, however, remain concerning the toxicity of Hg or the consequences of prehistoric Hg exposure on ecosystem and human health. Here, we show that Classic Period Hg concentrations in sediment cores from two lakes on the Yucatán Peninsula exceeded modern sediment Hg toxicity thresholds by orders of magnitude for centuries during ancient Maya occupation. Sediments also have evidence of aquatic food web collapse (algae, bacteria, cyanobacteria, ostracods, snails, and nonbiting midges), coincident with the time of high Hg concentrations, confirming that Hg toxicity was an additional environmental stressor for Classic Maya populations.

## INTRODUCTION

Mercury (Hg) is a toxic heavy metal capable of harming humans and ecosystems, even at low concentrations (*1*). Mercury is a neurotoxin that can enter the human body through inhalation, skin contact, and consumption of Hg-contaminated food and water (*2*). In aquatic ecosystems, elemental Hg can be converted into toxic methyl-Hg through bacterial transformation under anoxic conditions (*3*). Methyl-Hg and other forms of Hg in aquatic ecosystems can bioaccumulate in fish and other biota, thereby compromising environmental integrity and negatively affecting the health of humans who drink the lake water and consume aquatic protein (*4*). Despite these dangers, human release of Hg into the environment was widespread in the past and continues today, with modern releases from smelting, coal burning, industrial processes, and urban waste incineration. Ancient humans used elemental (liquid) Hg in amalgamation processes (*5*, *6*) as well as the mercury sulfide mineral cinnabar (HgS) as a decorative red pigment (*7*).

The ancient Maya of Mesoamerica were fascinated by Hg in both its elemental form (*8*, *9*) and as cinnabar, which they used as a pigment in burials and to decorate pottery and structures (*7*, *10*). Hg was shown to have reached toxic concentrations (1 μg g^−1^) at some localities (*5*, *11*, *12*), in association with buildings (*13*), soils (*14*, *15*), and reservoir sediments (*11*, *12*), but little is known about impacts to the ancient Maya or broader ecosystem health (*11*). It has been proposed that Hg in ancient Maya archaeological contexts originated from cinnabar and liquid Hg mined in volcanic regions of Honduras and/or central México (Teotihuacan) and was distributed through long-distance trade (*8*, *16*). However, the geographic extent of documented Hg use by the ancient Maya is limited largely to the southern highlands and south-central lowlands, with only a single occurrence in the northern Yucatán lowlands [Fig. 1; (*5*, *15*)]. Hg is generally associated with large urban centers such as Tikal, Quiriguá, and Kaminaljuyú in Guatemala, Copán in Honduras, and Caracol in Belize (*5*). Local watershed Hg transport is thought to have been associated with erosion of phosphorus-rich soils from the terrestrial realm and deposition in sediments of aquatic systems (*11*, *15*). Human exposure was believed to have occurred mostly through ritual use by high-status individuals (*11*), but it is now thought that some non-elite Maya experienced Hg exposure (*12*, *13*).

**Fig. 1.**
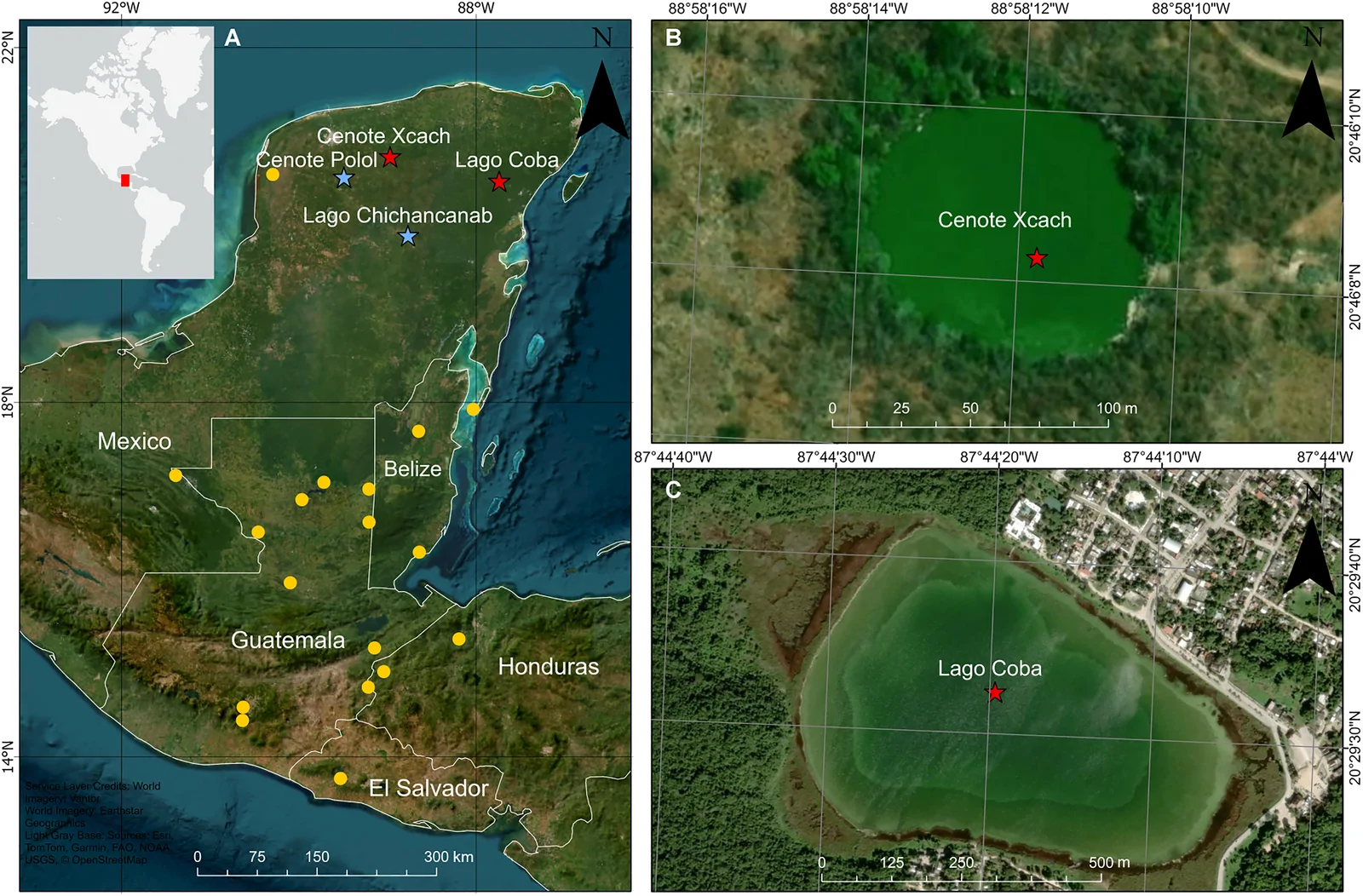
Map of Mesoamerica and study sites. (**A**) Map of the ancient Maya region showing the locations of Maya archaeological sites where Hg has been detected (yellow circles), our Hg-rich study lakes, Lago Cobá and Cenote Xcach (red stars), and reference lakes, Lago Chichancanab and Cenote Polol (blue stars). Hg site locations are based on Cook *et al.* (*5*). (**B** and **C**) Core locations in each lake are shown as red stars.

### Present study

We aimed to understand whether Hg was present at toxic levels in sediments of lakes in the Maya area, and if so, what impact Hg had on ecosystem structure and function. We analyzed sediment cores from two lakes (Lago Cobá and Cenote Xcach) on the northern Yucatán Peninsula, México, both of which had Pre-Columbian Maya structures nearby and lie far from areas of previously documented Hg contamination (Fig. 1). Cores were collected in May 2023 and chronologies (depth-age relationships) for the sequences were developed using AMS ^14^C dates (seven for Cobá and six for Xcach) from terrestrial macrofossils, fitted within a Bayesian dating model [(*17*); table S1 and fig. S1]. We sought to (i) measure total Hg concentrations and accumulation rates in the sediments, (ii) compare Hg concentrations to modern sediment quality guidelines (SQGs), and (iii) evaluate ecological impacts of Hg on aquatic biota in the lakes [bacteria, cyanobacteria, algae, ostracods, gastropods, and chironomids (nonbiting midges)] to assess biological consequences of Hg exposure and the potential for negative impacts to the ancient Maya.

Lago Cobá is a small (∼0.4 km^2^) shallow waterbody adjacent to the ruins of the ancient Maya urban center of Cobá (*18*). The watershed today hosts the modern town of the same name on the northeast and south shores of the lake. The ancient Maya established the site between Lago Cobá and its sister waterbody, Lago Macanxóc, which were important sources of water and aquatic protein for local inhabitants. Population at Cobá reached 50,000 to 90,000 during the Classic and Early Postclassic Periods (*18*, *19*), and the urban center included 35 raised roadways (*sacbeob*), two of which connected to other ancient Maya cities (*20*). Paleolimnological analyses of pollen, diatoms, and stable isotopes in a previously obtained sediment core from Lago Cobá revealed past changes in vegetation, aquatic biota, and climate conditions (*21*, *22*).

Our 2023 sediment core from Cobá is 6.4 m long and spans ∼7800 years of sediment accumulation. Here, we focus on the period from approximately 200 BCE to present, which encompasses the rise and fall of sediment Hg concentrations (figs. S2A and S3A). The 2200-year record closely matches the time span of the sediment core from Cenote Xcach, a small (∼100-m-diameter) sinkhole lake located adjacent to the minimally excavated Maya archaeological site of the same name. Cenote Xcach has at least four ancient-Maya civic-ceremonial structures nearby and is hypereutrophic today (fig. S4). The sediment core from Cenote Xcach is 5.8 m long and represents accumulation from approximately 100 BCE to present (fig. S1B and table S1).

## RESULTS

### Total mercury concentrations in the lake sediments through time

In the Lago Cobá core, total Hg concentrations began to increase above “background” values (∼0.02 μg g^−1^) approximately 40 CE, during the Late Preclassic period (Fig. 2), and continued to rise into the Classic period, reaching maxima ∼2.3 μg g^−1^ from approximately 250 to 300 CE. Concentrations then decreased steadily, returning to background values by approximately 520 CE, and remained <0.5 μg g^−1^ to the top of the core (2023 CE). Three periods of high Hg concentration were recorded in the Cenote Xcach sediment core. The first interval began around 125 CE and lasted until approximately 200 CE, with maximum concentrations around 3 μg g^−1^. The second episode of high Hg concentration occurred from 330 to 370 CE and reached a maximum value of ∼2.0 μg g^−1^, similar in both magnitude and timing to the Hg peak in the Lago Cobá core. The third episode of high Hg was the time of greatest metal concentrations, with largest values in the Cenote Xcach core measured in sediments deposited from approximately 535 to 820 CE, during which Hg averaged 8.4 ± 4.8 μg g^−1^ and registered a maximum value of ∼18 μg g^−1^ approximately 720 CE.

**Fig. 2.**
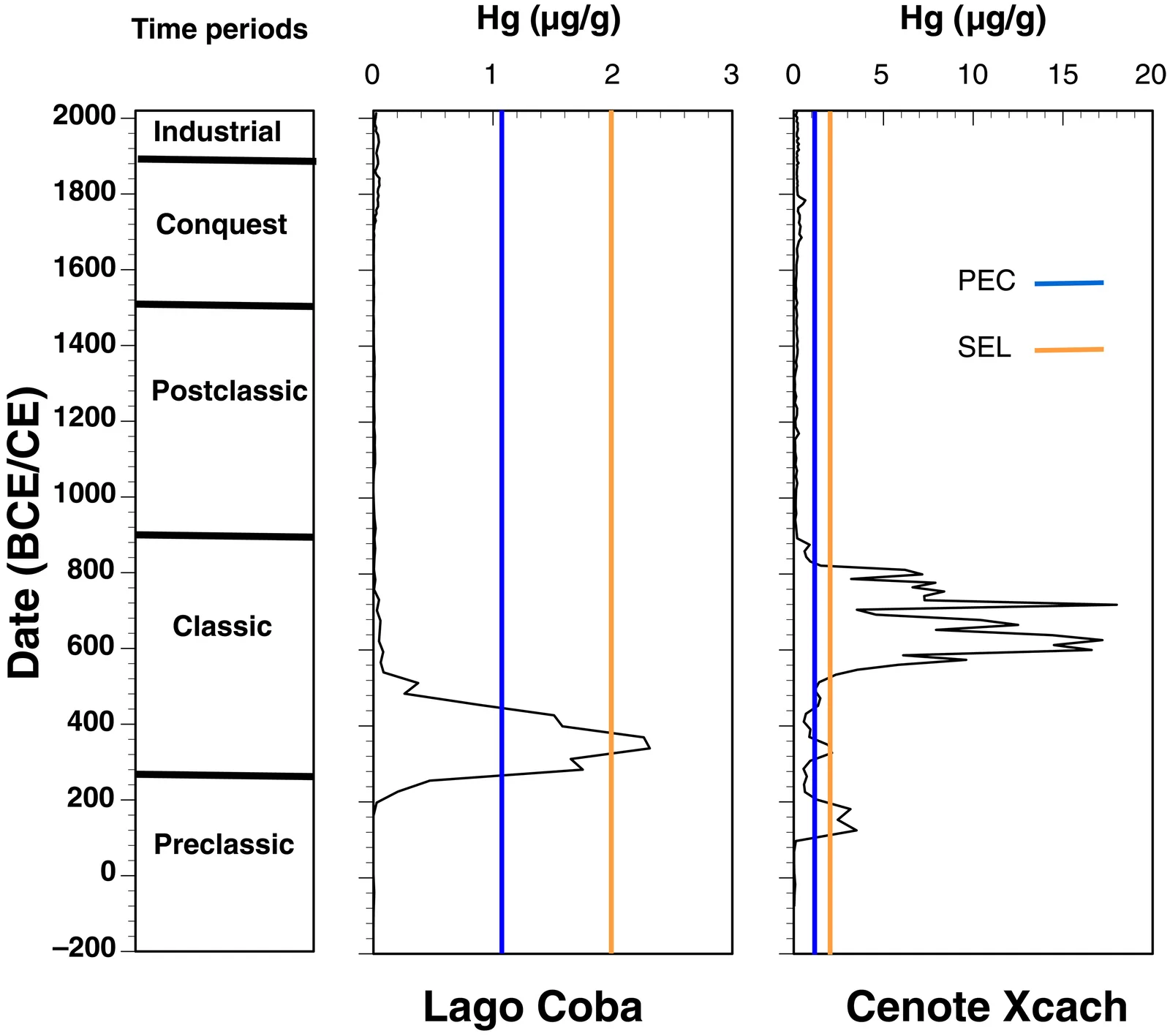
Total mercury results from Lago Cobá and Cenote Xcach sediment cores. Total mercury concentrations (μg g^−1^ dry) in the Lago Cobá and Cenote Xcach cores show values during the Maya Classic archaeological periods (left) exceeding consensus-based sediment quality guidelines for probable effect concentration [PEC, blue (1.06 μg g^−1^ dry)] (*24*) and severe effect level [SEL, orange (2 μg^−1^ dry)] (*25*).

We also measured Hg concentrations in sediment cores collected from two other northern Yucatán lakes during our visit in 2023, Polol and Chichancanab (Fig. 1). Neither core from those lakes showed elevated Hg concentrations, thus ruling out a widely distributed, atmospheric source for the heavy metal (fig. S2). We propose that the high Hg concentrations in the Cobá and Xcach sediments were a consequence of elemental Hg and/or cinnabar having been imported into the watersheds by the ancient Maya, after which it was transported into the lakes.

Total mercury concentrations in Classic Period sediments of Lago Cobá and Cenote Xcach were substantially greater than Hg values in sediments deposited during all other time periods (Table 1 and fig. S3). We calculated Hg enrichment factors (EFs) for each Maya archaeological period by normalizing sediment Hg concentrations to those of the stable element aluminum (Al). The method aligns with previous EF calculations and Hg studies in the Maya region, because Al is considered a conservative, abundant lithogenic element in Mesoamerica (*14*). Classic Period Hg EF values for Cobá and Xcach were 6× and 11× background crustal values, respectively, indicating that material that entered the lakes during the Classic Period had elevated Hg relative to typical, locally eroded material [(*23*); Table 1 and fig. S3]. Furthermore, Hg EF values <1 in other time periods indicate the absence of naturally occurring Hg in the soils of the Yucatán. Total Hg accumulation rates were calculated to remove the effect of changes in bulk sedimentation rate on Hg concentrations through time. Similar to EF values, Classic Period Hg accumulation rates in both lakes were 10× greater than during any other period (Table 1 and fig. S3). Total Hg concentrations, EFs, and accumulation rates all point to markedly greater Hg delivery to the two lakes during the Classic Period.

**Table 1.** Hg data for each historic period. Hg concentrations, enrichment factors, and accumulation rates for Lago Cobá and Cenote Xcach sediments dating to each Maya archaeological period, the conquest, and since the Industrial Revolution. Classic Period values are in bold. Values are the means (SD) for each time period. EF values were calculated after normalizing to background Al concentrations in each core during the Preclassic, following Turner *et al.* (*14*) and using the equation described in Materials and Methods. Hg accumulation rates (μg cm^−2^ year^−1^) were calculated from bulk density measurements (g dry cm^−3^ wet) and Hg concentrations (μg g^−1^ dry), with the calculated Hg in each cm^−3^ wet summed over the length of the core section spanning a time period, and divided by the number of years in that time period, computed from dates assigned to the top and bottom of the time period, determined from each core’s dating model (fig. S1 and table S1) as described in Materials and Methods.

	Time period	Core depth (cm)	Concentration (μg g^−1^)	EF	Accumulation rate (μg cm^−2^ year^−1^)
Lago Cobá	Industrial (1850 CE to present)	0–36	0.03 (0.01)	0.64 (0.18)	0.01 (0.01)
	Conquest (1520 to 1850 CE)	38–108	0.02 (0.02)	0.49 (0.28)	0.01 (0.01)
	Postclassic (920 to 1520 CE)	110–208	0.01 (0.01)	0.34 (0.20)	0.01 (0.01)
	**Classic (200 to 920 CE)**	**210–300**	**0.59 (0.81)**	**3.91 (3.91)**	**0.11 (0.15)**
	Preclassic* (200 BCE to 200 CE)	302–336	0.02 (0.06)	0.15 (0.35)	0.01 (0.01)
Cenote Xcach	Industrial (1850 CE to present)	0–66	0.20 (0.04)	0.48 (0.14)	0.03 (0.03)
	Conquest (1520 to 1850 CE)	68–188	0.24 (0.11)	0.48 (0.16)	0.10 (0.06)
	Postclassic (920 to 1520 CE)	190–340	0.01 (0.05)	0.51 (0.37)	0.05 (0.02)
	**Classic (200 to 920 CE)**	**342–500**	**5.25 (5.18)**	**5.69 (4.92)**	**2.07 (2.37)**
	Preclassic* (200 BCE to 200 CE)	502–588	0.92 (1.29)	0.85 (1.02)	0.18 (0.23)

* Preclassic period begins with the basal date of Cenote Xcach and the corresponding date for the Lago Cobá core.

We used modern SQGs to assess the toxicity of Hg concentrations in the Lago Cobá and Cenote Xcach sediment cores. Consensus-based SQGs for mercury include a probable effect concentration (PEC) of 1.06 μg g^−1^, at which harmful effects are likely to occur (*24*), and a severe effect level (SEL) of 2.0 μg g^−1^, at which extreme impacts are expected for biota (*25*). In Lago Cobá, PEC values were exceeded during the Early Classic period, approximately 260 to 430 CE, with SEL values exceeded from about 340 to 370 CE (Fig. 2). Those concentrations occurred when agricultural activity at Cobá moved away from the city center and urbanization around the lake increased (*18*, *21*). The Hg increase in the Lago Cobá core preceded archaeological evidence for maximum urban population florescence, as suggested by the erection of stelae only during the Late Classic, from about 500 to 800 CE (*26*). In the Cenote Xcach core, there are three periods of toxic Hg concentrations. The first occurred from about 125 to 180 CE and the second coincided with the PEC levels in the Lago Cobá sediments, from approximately 330 to 350 CE. The third period of Hg concentration in Cenote Xcach was of greater magnitude and duration than the first two. During the third period, SEL values were first exceeded approximately 535 CE and remained above 2.0 μg g^−1^ until 810 CE, displaying extreme toxic Hg concentrations for several centuries (Fig. 2). Values during this third period were frequently >10.0 μg g^−1^, with a maximum value of 18.0 μg g^−1^ around 720 CE. Given the paucity of archaeological information from the Cenote Xcach watershed, we cannot yet link these high sediment Hg values to a specific phase of urban development in the area.

### Hg impacts on aquatic biota

Whereas recognition of Hg occurrence at ancient Maya sites is not new (*5*, *27*), sustained concentrations above modern SQGs like those in the Lago Cobá and Cenote Xcach sediments have not been documented and point to Hg toxicity capable of affecting aquatic biota and, likely, human health. In both systems, sediment concentrations of diagnostic photosynthetic pigments for total primary producer abundance (beta-carotene), cyanobacteria (echinenone at Cobá and canthaxanthin at Xcach), and purple-sulfur bacteria (okenone) (*28*) declined substantially to minimal levels once toxic Hg concentrations were reached (Fig. 3). In Lago Cobá, inferred total abundances of primary producers and purple sulfur bacteria were low at the beginning of the high-Hg period, but bioindicators of cyanobacteria increased, possibly in response to greater total phosphorus (TP) inputs. Nevertheless, the cyanobacteria community collapsed soon after SEL values for Hg were reached (Fig. 3). Similarly, during the period of highest Hg in the Cenote Xcach core, when Hg concentrations greatly exceeded those in the Lago Cobá core, values for most pigment markers initially increased alongside greater TP inputs, but then all three primary producer groups collapsed soon after SEL levels of Hg were surpassed (Fig. 3).

**Fig. 3.**
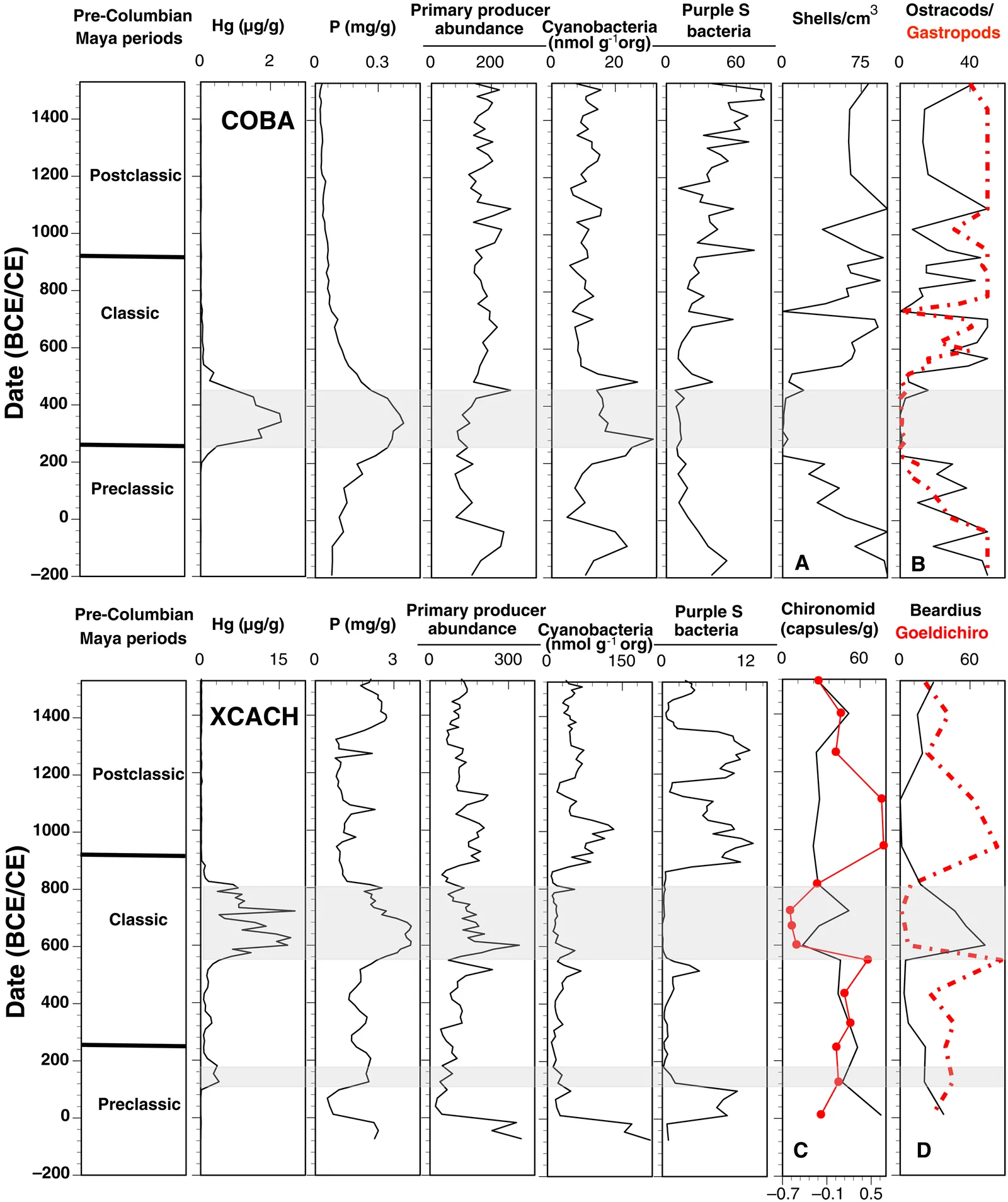
Paleolimnological proxy data from the Lago Cobá and Cenote Xcach sediment cores. Paleolimnological variables versus dates for the sediment cores from Lago Cobá and Cenote Xcach. Intervals when Hg concentrations exceeded SEL thresholds (gray bars) occur during the Preclassic and Classic. Maya archaeological periods (left). Total mercury (Hg) is reported as μg g^−1^ dry, and total phosphorus is reported as mg g^−1^ dry. Photosynthetic pigments for primary producer abundance (beta-carotene), cyanobacteria (echinenone for Coba and canthaxanthin for Xcach), and purple sulfur bacteria (okenone) are reported as nmol per g^−1^ organic matter. Pigment associations are based on Leavitt and Hodgson (*28*). For the Lago Cobá core, total gastropod and ostracod shells are reported as combined counts up to 50 per cm^−3^ wet sediment for each shell type (i.e., 100 total) (**A**) and separated as ostracods (solid black line) and gastropods (dashed red line) reported as counts up to 50 per cm^−3^ wet sediment (**B**). Chironomid head capsules are shown as numbers per gram of wet sediment and unitless values for principal component 1 from principal components analysis of all capsules analyzed (in red) (**C**). Total chironomid head capsules per gram of sediment are shown for *Beardius* spp. (black) and *Goeldichironomus carus* type (red) (**D**).

Mercury mobilized across Maya landscapes along with phosphorus, probably associated with enhanced soil erosion (*15*). Stratigraphic correlation between Hg and TP in the Lago Cobá and Cenote Xcach cores supports this proposed mode of transport, as do similar stratigraphic patterns for S and Al (Fig. 3 and fig. S5). Organic carbon, also known to bind to Hg, did not show stratigraphic patterns similar to Hg [fig. S5, (*29*)]. However, organic carbon values were high in both cores throughout the ancient Maya periods with Xcach and Coba averaging 21.3% (±5.7) and 15.5% (±1.4), respectively. Phosphorus is often the limiting nutrient for aquatic primary production and both limnological and paleolimnological studies have shown that increased phosphorus inputs to lakes often drive greater algal and cyanobacterial productivity (*30*–*33*). Increased TP inputs to our study lakes initially triggered greater cyanobacteria proliferation. Despite sustained TP inputs, that proliferation was followed by marked decreases in those primary producers, which we attribute to toxic Hg concentrations in the lakes.

Declines in indicators of primary producers and bacteria in the two basins suggest disruption of aquatic food webs, likely due to toxic Hg concentrations. That inference is supported by microfossil remains of organisms from higher trophic levels. In the Lago Cobá core, the only core interval without calcareous ostracod and gastropod shells was associated with toxic Hg concentrations (Fig. 3, A and B; numbers per cubic centimeter of wet sediment declined to 0). The interval devoid of shells from benthic invertebrates was noted but unexplained in a previously analyzed Lago Cobá sediment core (*22*), because Hg had not been measured. Cenote Xcach sediments lack calcareous shells, so chitinous head capsules of chironomid larvae were counted throughout the core. Head capsule numbers declined with the initial increase in Hg concentration (Fig. 3C). Alongside changes in total head capsule abundance, that period is characterized by a complete compositional turnover of chironomid genera, shown as a negative excursion in PC axis 1 (Fig. 3C and fig. S6). During the high-Hg period, the previously dominant chironomid genus *Goeldichironomus* nearly vanished and was replaced almost entirely by the genus *Beardius* (Fig. 3D).

## DISCUSSION

High concentrations of Hg in Classic Period sediments from Lago Cobá and Cenote Xcach provide circumstantial evidence for Maya trade and use of liquid mercury and/or cinnabar, and potential Hg toxicity at some locations. Lentz *et al.* (*11*) reported high Hg concentrations in sediments from reservoirs at Tikal and proposed that ruler “Dark Sun” may have suffered health consequences from Hg exposure, including metabolic syndrome and obesity. Hg concentrations in soils around the Marco Gonzalez site in Belize (*14*) and in reservoir sediments at the Maya city of Ucanal, Guatemala, also exceeded PEC values (*12*). Elemental Hg was found at the archaeological sites of Lamanai, Belize (*10*) and Copán, Honduras (*8*). Although evidence of mercury contamination extends throughout Mesoamerica, concentrations in sediments at only a few sites exceeded PEC and SEL values or show persistent high Hg concentrations, as in our cores from Lago Cobá and Cenote Xcach (*5*, *11*).

### Implications for the ancient Maya

Toxic concentrations of Hg in sediments from both study lakes, along with evidence for impacts across multiple trophic levels in the waterbodies, indicate that Hg contamination caused an aquatic ecosystem collapse that coincided with Classic Period Maya occupation. Cinnabar (HgS) is a relatively recalcitrant form of Hg, with the greatest adverse impacts to biota occurring after transformation into methyl-Hg, not measured here. Methyl-Hg genesis, however, is common in benthic areas of lakes, where anoxia, organic matter, and bacteria combine to create ideal conditions for methyl-Hg production (*4*, *34*–*37*).

Multiple, independent biological proxy variables support the movement of Hg into aquatic food webs. Declines in biomarker concentrations for cyanobacteria (*3*), total primary producer abundance (*38*), and purple sulfur bacteria (i.e., okenone) collectively indicate primary producer disruption during periods of peak Hg input (*39*). At higher trophic levels, the coeval disappearance of ostracod and gastropod shells in Lago Cobá plus marked reductions and taxonomic changes in chironomid head capsules in Cenote Xcach further demonstrate the ecological consequences of Classic Period Hg toxicity (Fig. 3). Collectively, synchronicity of these biological responses with peak Hg concentrations suggests ecosystem-wide stress beyond taxon-specific effects and a bioaccumulation of Hg throughout the aquatic biota.

As Hg accumulated across multiple levels of the aquatic food web, ancient Maya populations living near these water bodies would conceivably have been exposed through consumption of water and lake-derived protein. Fish and shellfish are known components of the ancient Maya diet (*40*), and both groups are efficient bioaccumulators of mercury (*41*, *42*). It is therefore possible that human populations at Cobá and Xcach experienced chronic Hg exposure for decades to centuries at concentrations considered toxic by modern SQGs.

Hg concentrations that exceed SEL are rarely seen in sediments of undisturbed (natural) systems, and ancient societal impacts and human responses to such environmental changes have rarely been addressed. Lentz *et al.* (*11*) linked Hg poisoning with obesity, expressed in ancient Maya artistic depictions of nobles, and there is also evidence of Hg in human bones from the Maya region (*7*, *43*, *44*). In addition, elemental Hg has been reported from nine Maya archaeological sites, and soils and sediments from six other sites had Hg concentrations above PEC levels established for human health [(*5*) and references therein]. Whereas climate-associated reductions in water quantity are well established as a regional stressor for the ancient Maya (*45*–*47*), our results suggest that declining water quality driven by Hg contamination constituted an additional and previously underappreciated environmental stressor during the Classic Period.

## MATERIALS AND METHODS

### Core collection

Sediment cores from Lago Cobá, Cenote Xcach, Cenote Polol, and Laguna Chichancanab were collected using a mud-water interface corer (*48*) for the top 80 cm and a modified Livingstone piston corer for subsequent 1-m sections, beginning at 50-cm sediment depth. Core sections included a primary core site and an adjacent site for overlapping cores (50-cm depth offset) to ensure a complete sediment record was retrieved. Permission for sediment collection was granted by I. Pech, Delegado Municipal de Cobá for Lago Cobá; L. C. Chan, landowner for Cenote Xcach; E. S. Loeza, the landowner for Cenote Polol; and U. Ku y Chi, Comisario Ejidal de Dziuche, Quintana Roo, for Laguna Chichancanab. The coring location for Lago Cobá was near the center of the lake (Fig. 1), close to where a previous core was taken (*21*). Water depth at the coring site was 4.2 m and total core length was 6.41 m. For Cenote Xcach, the coring site was chosen following a brief bathymetric survey of the lake, and a presumed depositional area was targeted for coring. Water depth at the coring site was ∼10 m and a 5.88-m-long core was retrieved. The mud-water interface cores were extruded vertically in the field in 2-cm sections and stored in plastic Whirl-Pak bags. Deeper core sections were left in their polycarbonate core barrels and returned intact to the laboratory for analyses.

### AMS ^14^C dating and age-depth model

For the Lago Cobá core, six charcoal samples and one terrestrial wood fragment were collected from the core for AMS ^14^C dating (table S1). Charcoal samples were pretreated for analysis using a 2-day KOH/H_2_O_2_ wash. The remaining sediment was washed over a 0.125-mm sieve, and charcoal fragments were collected using a dissecting microscope at 40× magnification. For the Cenote Xcach core, two charcoal and four terrestrial wood fragments were collected throughout the core and processed as described above for the Cobá samples. Radiocarbon dating was carried out at the National Ocean Sciences Accelerator Mass Spectrometry Laboratory at the Woods Hole Oceanographic Institution. To construct depth-age relationships for each core, all uncalibrated AMS ^14^C ages were incorporated into the Bayesian dating model Bacon (*17*) in R v 4.5.0 (fig. S1). The dating model assigned a date to every centimeter depth in each core.

### Laboratory analyses

All chemical measurements were performed on sediment subsamples that were freeze-dried and ground with a mortar and pestle. Organic carbon and total nitrogen were measured using a Costech ECS 4010 elemental combustion analyzer after weighing dried samples into silver or tin capsules, respectively. For organic carbon, weighed samples were acidified in an HCl vapor chamber for 24 hours before analysis to remove inorganic (carbonate) carbon. Acidified samples were then placed into tin capsules to promote thorough combustion in the analyzer. All other elements including total Hg were analyzed at Waters Agricultural Laboratories Inc. in Camilla, GA, USA (https://watersag.com). Inductively coupled plasma spectroscopy was used to analyze all elements except C, N, and Hg, after acid digestion (HCl and HNO_3_) in a heated block, following Environmental Protection Agency (EPA) method 6010B. Blanks and QC checks were included in every set, and a North American Proficiency Testing Program check standard for soil analysis was run after every 40 samples. Total mercury was measured using a DMA-80 Direct Hg Analyzer following EPA method 7473, which uses a direct injection of dried sediment without the need for extraction. Check standards for the Hg analysis were 1, 5, 10, and 18.2 μg g^−1^, representing the range of values in our sediment samples. Each check standard was run twice during each run as well as triplicate samples four times during the runs.

Photosynthetic pigments (chlorophylls and carotenoids) were analyzed on a Shimadzu HPLC system within 30 days of freeze-drying to reduce degradation. Pigments were extracted from weighed sediment samples using a solvent mixture of 80% acetone, 15% methanol, and 5% HPLC-grade water that included an internal standard (Apocarotenal or trans-β-apo-8′-carotenal; Sigma-Aldrich Chemical Corporation). Extractions occurred for 16 to 24 hours in a −20°C freezer followed by centrifugation. The supernatant was removed and passed through a 0.22-µm syringe filter to remove particulate material (*30*). Pigment samples were injected into the HPLC system and separated by passing through a Phenomenex Luna C18 column following the mobile phase and time sequence of Leavitt and Hodgson (*28*) and Waters *et al.* (*49*). Carotenoids and chlorophylls were determined based on spectra obtained from a PDA detector for carotenoids and a fluorescence detector for chlorophylls. Concentrations were determined by comparing peak area and retention times to known standards for each pigment (DHI, Denmark) and reported as nmol per g^−1^ organic matter.

Ostracod and gastropod samples from the Lago Cobá core were prepared following Whitmore *et al.* (*22*). Briefly, 4.92 cm^3^ (1 teaspoon) of wet sediment was disaggregated using 35% hydrogen peroxide and sieved to remove clay and silt particles. Samples were transferred to a petri dish and ostracods and gastropods were enumerated under a dissecting microscope at 10× magnification. Whole ostracod and gastropod shells were counted within each sediment interval up to a maximum of 50 individuals per taxonomic group. In samples for which shell abundance exceeded this threshold, counts were capped at 50 to standardize counting effort across samples. In samples containing fewer than 50 shells, all individuals were counted. Here, shell counts were used to assess relative changes in numbers of each taxon through time, rather than absolute population sizes or shifts in relative abundances between ostracods and snails. There were no ostracods or gastropods in the Cenote Xcach core, so wet sediment samples (1.3 to 2.0 g) were collected for chironomid analysis and prepared according to Brooks *et al.* (*50*) using potassium hydroxide (10%), sieving, and sonicating for three 5-s pulses. All identified head capsules were picked from the sieved material and mounted using a Nikon SMZ1000 low-power microscope. Fossil chironomid head capsules were identified using a Brunel Microscopes SP150 high-power binocular microscope at 400× magnification using Wiederholm (*51*) and Hamerlík *et al.* (*52*) as reference material. Principal components analysis was performed in R using the “Vegan” package (*53*).

### EF values and accumulation rate calculations

EFs for Hg were calculated for the Lago Cobá and Cenote Xcach cores following the equation from Turner *et al.* (*14*)

$${\text{EF}}_{\text{Hg}}=[\left({\text{Hg}}_{\text{sample}}/{\text{Al}}_{\text{sample}}\right)/\left({\text{Hg}}_{\text{background}}/{\text{Al}}_{\text{background}})\right]$$

with background values represented by the mean value of each element in the Preclassic, before the onset of elevated Hg values. Mean Hg accumulation rates (μg cm^−2^ year^−1^) were calculated for each time period following Vachula *et al.* (*54*). That was accomplished by multiplying the Hg concentration (μg g^−1^ dry) in each centimeter slice within a time period by its corresponding dry bulk density (g dry cm^−3^ wet), yielding the amount of Hg in each wet cubic centimeter of sediment throughout the time period. Those values were then summed to obtain the total amount of Hg beneath a square centimeter in the period (μg cm^−2^), and that value was divided by the total number of years in the time period to get μg Hg cm^−2^ year^−1^.
