# Supplementary material for: Toxic mercury, aquatic ecosystem collapse, and the ancient Lowland Maya
Source: Sci Adv. 2026 Nov 4;12(45):eaeh3280. doi: 10.1126/sciadv.aeh3280 (PMC13733889; doi:10.1126/sciadv.aeh3280)
Supplement: Supplementary file 1 [file sciadv.aeh3280_sm.pdf]

Science Advances
AAAS

Supplementary Materials for

Toxic mercury, aquatic ecosystem collapse, and the ancient Lowland Maya

Matthew Neal Waters *et al.*

Corresponding author: Matthew Neal Waters, mnw0018@auburn.edu

Sci. Adv. **12**, eaeh3280 (2026)
DOI: 10.1126/sciadv.aeh3280

This PDF file includes:

Figs. S1 to S6
Table S1

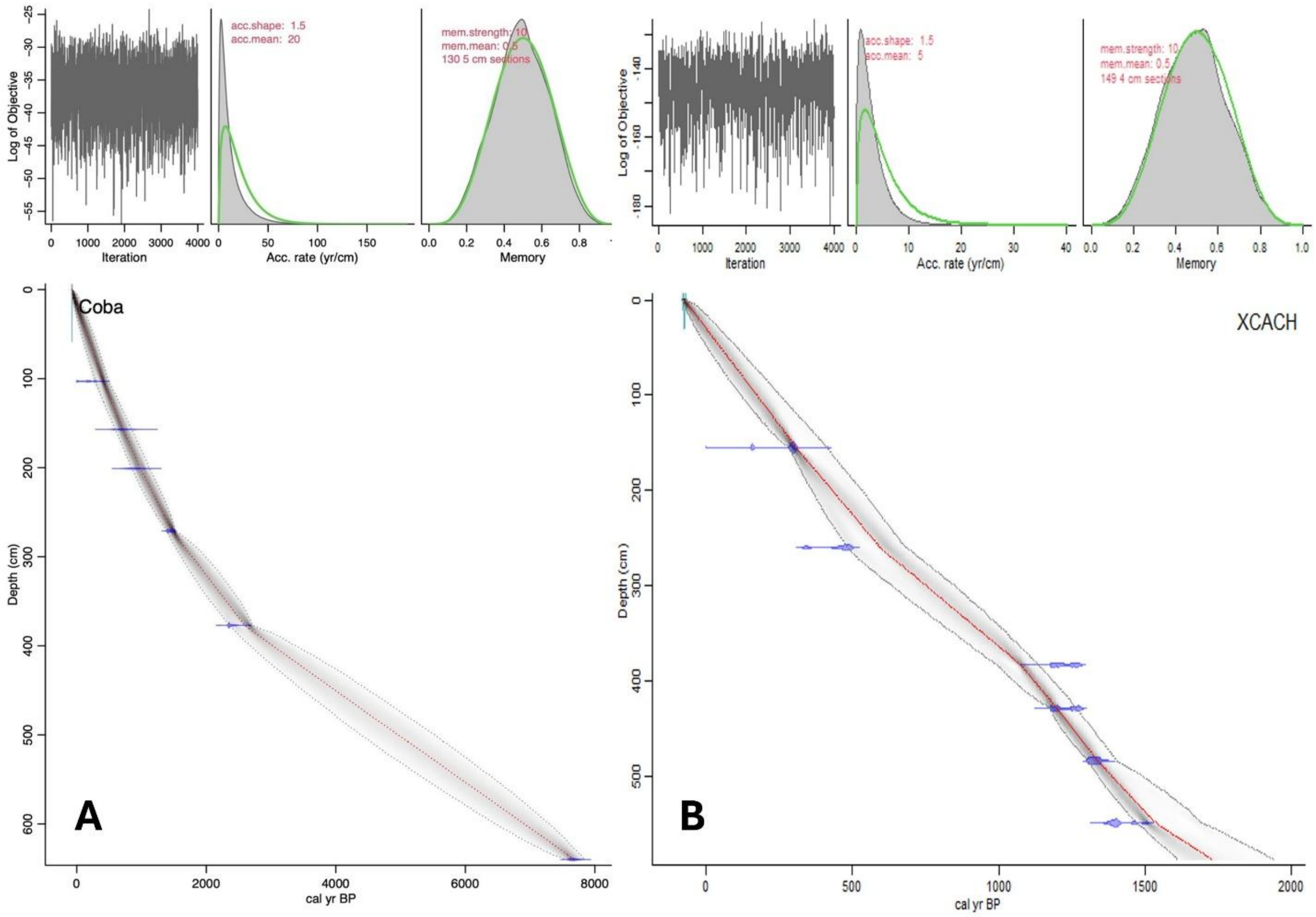

Figure S1. Bayesian Bacon dating model for the Lago Cobá (A) and Cenote Xcach (B) sediment cores, using the calibrated AMS^{14}C dates from Table S1 and the year 2023 CE for the top of each sediment core. Dating models were generated in R using the Bacon package (*17*).

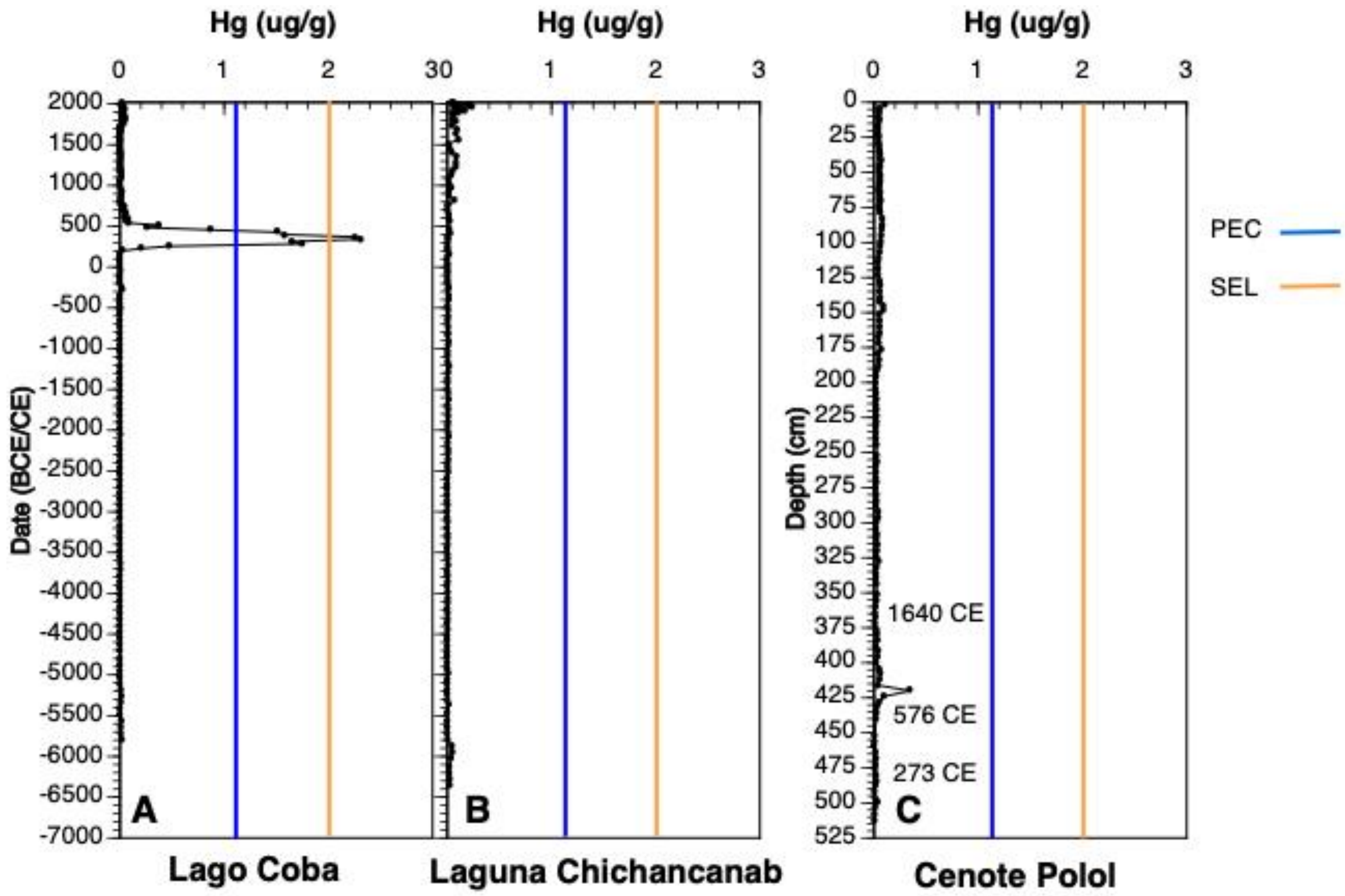

Figure S2. Total mercury concentrations (µg g^{-1} dry) vs. dates for the entire Lago Cobá and Laguna Chichancanab sediment cores (A and B). Total mercury concentrations vs. depth for Cenote Polol with calibrated dates for three ^{14}C AMS dates on charcoal material (C). Consensus-based sediment quality guidelines for probable effect concentration (PEC, blue [1.06 µg g^{-1} dry]) (*24*) and severe effect level (SEL, orange [2 $µg^{-1}$ dry]) (*25*).

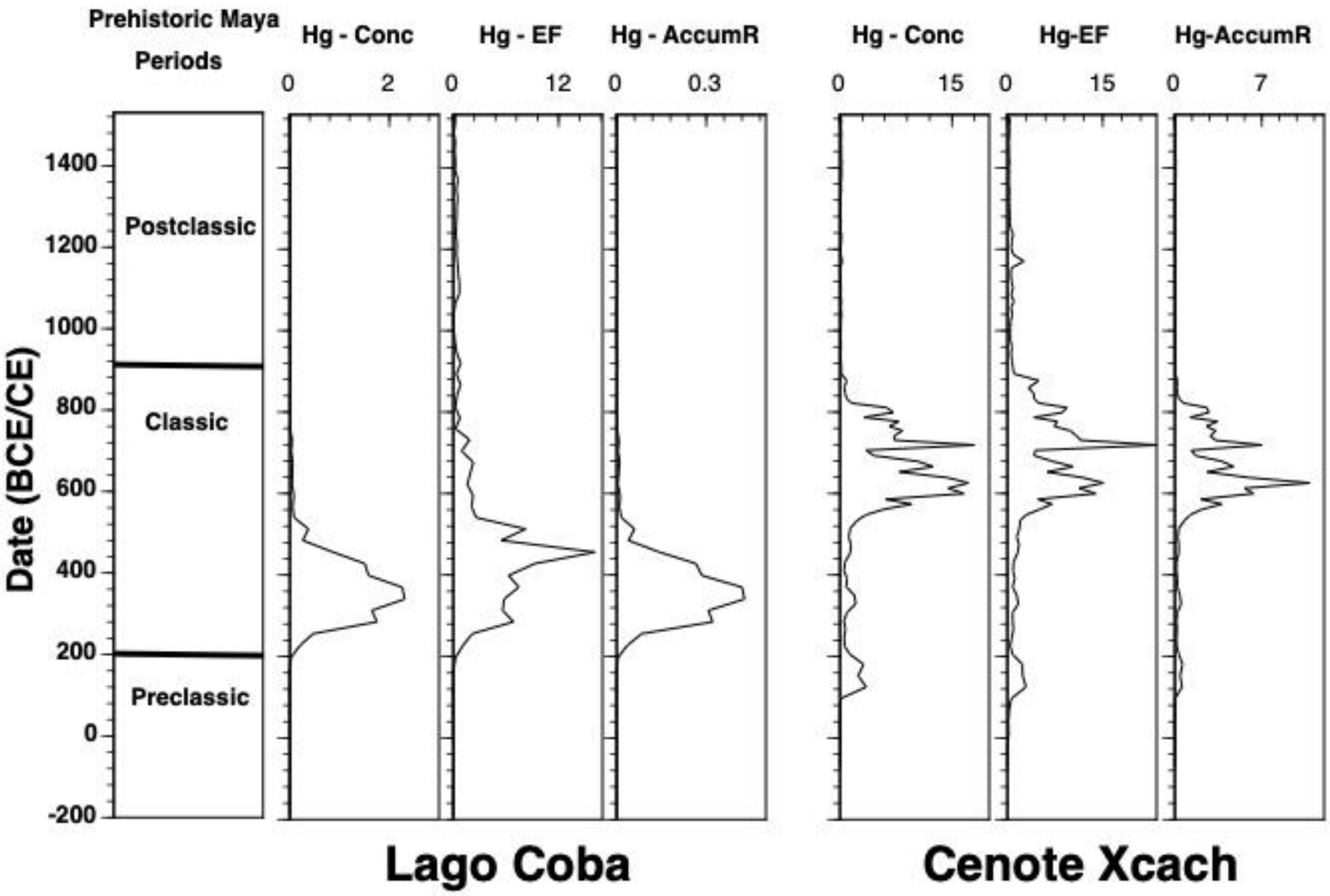

Figure S3. Total mercury concentrations (μg g^{-1} dry), enrichment factors (EF) and accumulation rates (μg cm^{-2} yr^{-1}) vs. dates for the Lago Cobá and Cenote Xcach cores. Left column shows Maya archaeological periods.

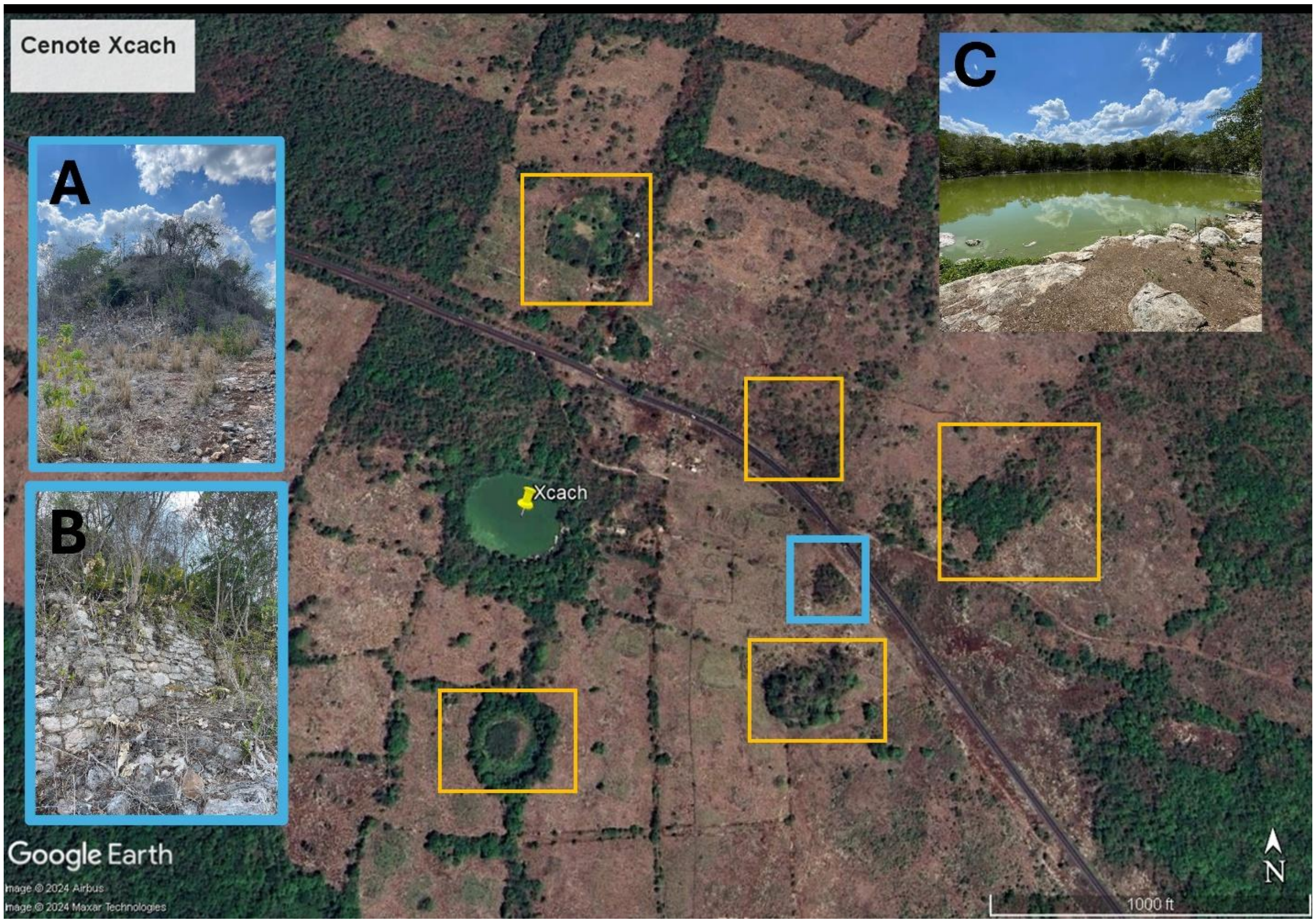

Figure S4. Google Earth image of Cenote Xcach. Yellow boxes show unexcavated structures with pictured structure in a blue box. Inset photos show the structure (A) and stonework (B) of one structure (blue box). Additional photo shows Cenote Xcach during fieldwork in 2023 CE. Map by Google Earth and image provided by Airbus (2024) and Maxar (2024). Inset photos (A,B,C) by Matthew Waters.

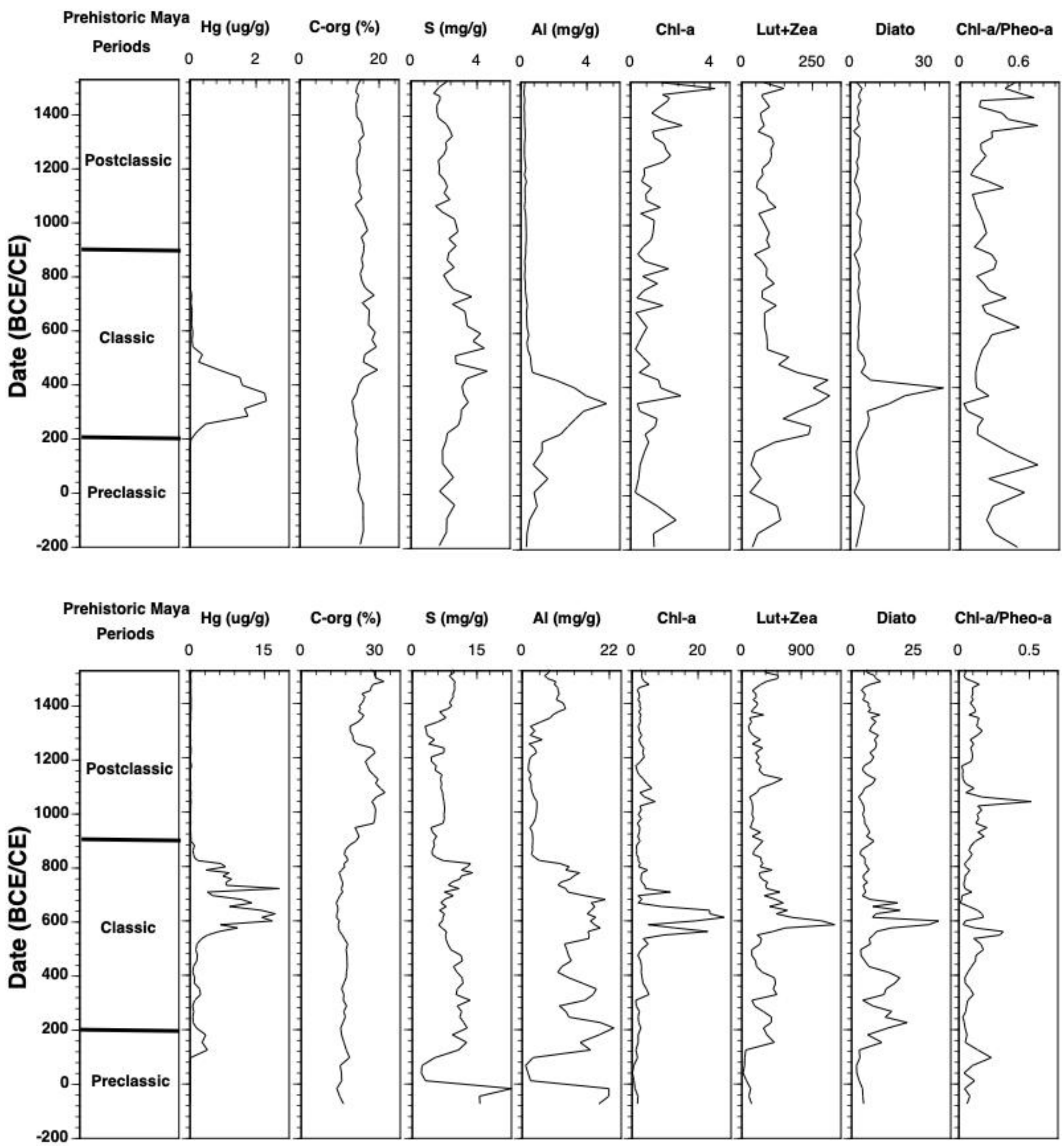

Figure S5. Additional sediment variables versus dates for the sediment cores from Lago Coba (upper plot) and Cenote Xcach (lower plot). Total mercury (Hg) is reported as $\mu g\ g^{-1}$ dry sediment, organic carbon (C-org) is reported as percent of dry sediment, and sulfur and aluminum are reported as $mg\ g^{-1}$ dry sediment. Photosynthetic pigments for primary producer abundance (Chlorophyll a), cyanobacteria + chlorophytes (Lutein+Zeaxanthin) and diatoms (Diato) are reported as $nmol\ g^{-1}$ organic matter. Pigment associations are based on Leavitt and Hodgson (*28*). The ratio Chlorophyll-*a*/Pheophytin-a is a degradation index and is unitless, with higher values indicating better pigment preservation.

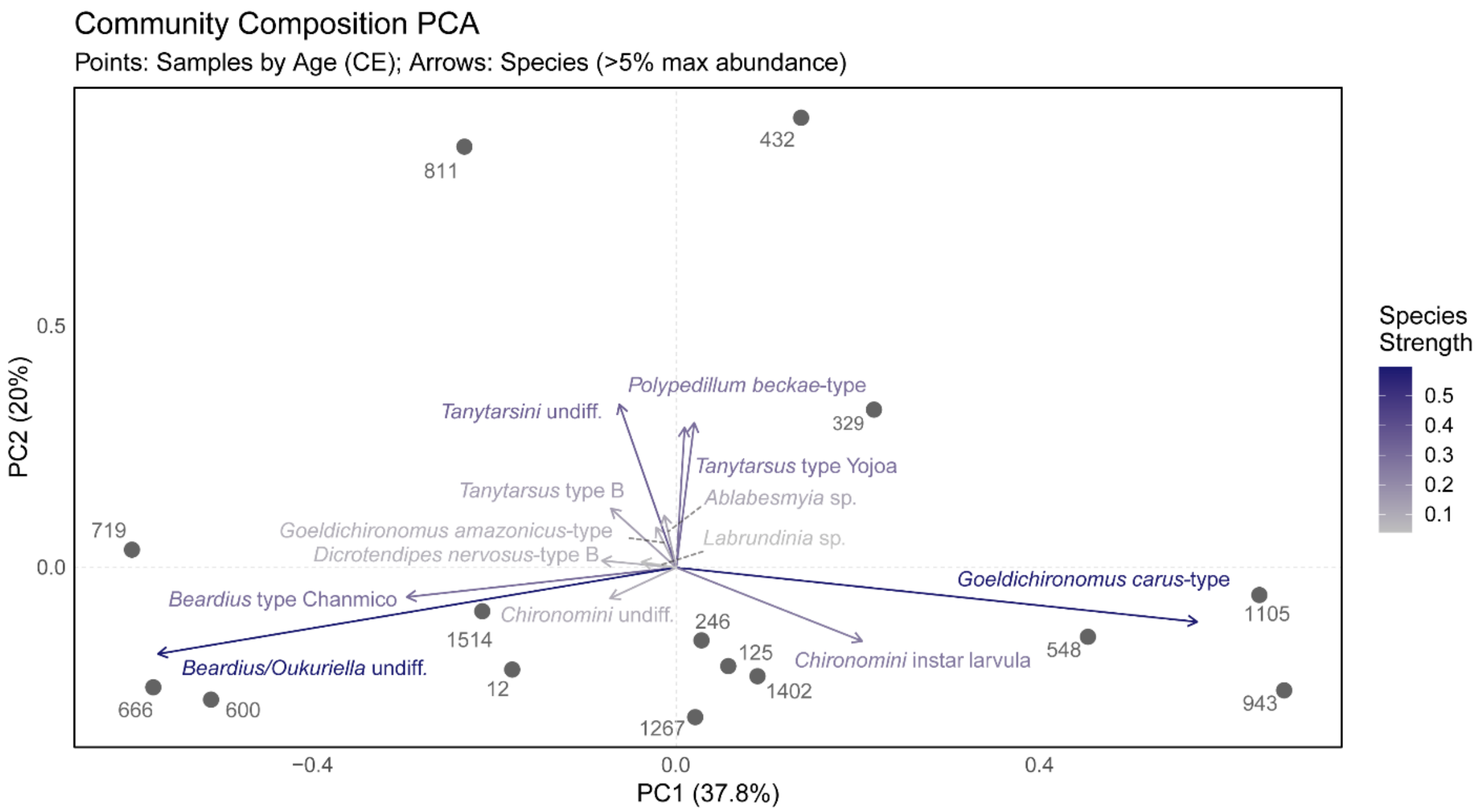

Figure S6. Scatterplot of principal components 1 (PC1) and 2 (PC2) for chironomid measurements from the Cenote Xcach sediment core. PC1 and PC2 accounted for 37.8% and 20% of the variance of the data-set, respectively. Species strength was determined by number of head capsules per chironomid type in the entire dataset. Stratigraphy of PC1 values is shown in Fig. 3C.

Table S1. Information for AMS ^{14}C dates. AMS ^{14}C dates, and calibrated dates used in the dating models for each core.

Lago Cobá

Depth (cm)	^{14}C Age (BP)	^{14}C Age Error (yrs)	Calib Age (CE/BCE)	Calib Age Range	Material Measured	Lab #
**103**	210	50	1763	1713-1813	Charcoal	190502
**157**	750	110	1254	1115- 1408	Charcoal	190503
**201**	990	90	1066	882- 1230	Charcoal	190504
**271**	1,560	20	493	472- 567	Plant/Wood	188126
**314**	1,830	15	223	200- 248	Charcoal	198655
**377**	2,350	30	408 BCE	517- 380 BCE	Charcoal	190505
**640**	6,840	50	5722 BCE	5831- 5634 BCE	Charcoal	188131

Cenote Xcach

Depth (cm)	Age (BP)	Age Error	Calib Age (CE)	Calib Age Range	Material Measured	Lab #
**155**	250	15	1653	1640-1665	Plant/Wood	175499
**260**	395	20	1473	1446-1509	Plant/Wood	175500
**383**	1290	15	728	668-774	Plant/Wood	175501
**429**	1300	15	739	664-773	Charcoal	184979
**484**	1440	15	621	599-647	Charcoal	184980
**549**	1540	15	550	533-589	Plant/Wood	175502
